# Cardiac Computed Tomography Measurements in Pulmonary Embolism Associated with Clinical Deterioration

**DOI:** 10.5811/westjem.20763

**Published:** 2025-01-15

**Authors:** Anthony J. Weekes, Angela M. Pikus, Parker L. Hambright, Kelly L. Goonan, Nathaniel O’Connell

**Affiliations:** * Atrium Health’s Carolinas Medical Center, Department of Emergency Medicine, Charlotte, North Carolina; † Wake Forest School of Medicine, Department of Biostatistics and Data Science, Winston-Salem, North Carolina

## Abstract

**Introduction:**

Most pulmonary embolism response teams (PERT) use a radiologist-determined right ventricle to left ventricle ratio (RV:LV) cut-off of 1.0 to risk-stratify pulmonary embolism (PE) patients. Continuous measurements from computed tomography pulmonary angiograms (CTPAs) may improve risk stratification. We assessed associations of CTPA cardiac measurements with acute clinical deterioration and use of advanced PE interventions.

**Methods:**

This was a retrospective study of a PE registry used by eight affiliated emergency departments. We used an artificial intelligence (AI) algorithm to measure RV:LV on anonymized CTPAs from registry patients for whom the PERT was activated (2018–2023) by institutional guidelines. Primary outcome was in-hospital PE-related clinical deterioration defined as cardiac arrest, vasoactive medication use for hypotension, or rescue respiratory interventions. Secondary outcome was advanced intervention use. We used bivariable and multivariable analyses. For the latter, we used least absolute shrinkage and selection operator (LASSO) and random forest (RF) to determine associations of all candidate variables with the primary outcome (clinical deterioration), and the Youden index to determine RV:LV optimal cut-offs for primary outcome.

**Results:**

Artificial intelligence analyzed 1,467 CTPAs, with 88% agreement on RV:LV categorization with radiologist reports (kappa 0.36, 95% confidence interval [CI] 0.28–0.43). Of 1,639 patients, 190 (11.6%) had PE-related clinical deterioration, and 314 (19.2%) had advanced interventions. Mean RV:LV were 1.50 (0.39) vs 1.30 (0.32) for those with and without clinical deterioration and 1.62 (0.33) vs 1.35 (0.32) for those with and without advanced intervention use. The RV:LV cut-off of 1.0 by AI and radiologists had 0.02 and 0.53 *P*-values for clinical deterioration, respectively. With adjusted LASSO, top clinical deterioration predictors were cardiac arrest at presentation, lowest systolic blood pressure, and intensive care unit admission. The RV:LV measurement was a top 10 predictor of clinical deterioration by RF. Optimal cut-off for RV:LV was 1.54 with odds ratio of 2.50 (1.85, 3.45) and area under the curve 0.6 (0.66, 0.70).

**Conclusion:**

Artifical intelligence-derived RV:LV measurements ≥1.5 on initial CTPA had strong associations with in-hospital clinical deterioration and advanced interventions in a large PERT database. This study points to the potential of capitalizing on immediately available CTPA RV:LV measurements for gauging PE severity and risk stratification.

Population Health Research Capsule What do we already know about this issue?

*Pulmonary embolism (PE) response teams focus on patients with right ventricular dysfunction using CT findings of right ventricle to left ventricle (RV:LV) ratio of 1.0 or greater.*

What was the research question?

*What CT RV:LV measurements are associated with acute clinical deterioration?*

What was the major finding of the study?

*The optimal cut-off for RV:LV on CT was 1.54 with an odds ratio of 2.50 (1.85–3.45) for acute clinical deterioration.*

How does this improve population health?

*A RV:LV threshold of 1.5 on CT may improve PE risk stratification and inform use of inpatient resources.*

## INTRODUCTION

Established pulmonary embolism (PE) risk-stratification guidelines employ binary assessments of hemodynamic stability and right ventricular dysfunction (RVD) using imaging modalities and troponin.^1^ The main imaging modalities of RVD are echocardiography and computed tomography pulmonary angiogram (CTPA). Comprehensive echocardiography provides multifaceted RVD assessments; however, it rarely confirms diagnosis of PE and may not be immediately available. A CTPA diagnoses PE and identifies limited parameters of RVD, usually as right ventricle (RV) dilatation. Radiologists usually report on RVD as a binary variable of RV to left ventricle diameter ratio (RV:LV) using a range of cut-offs from 0.9 to 1.5.^2^ ^–^ ^7^ Right ventricular dysfunction on CTPA, when expressed as a continuous variable, may be a better predictor than its binary version.

Consistent reporting of RVD measurements may be labor intensive for radiologists. Artificial intelligence (AI) algorithms have been developed to assist radiologists’ workflow by simultaneously interpreting presence of filling defects and measuring cardiac chamber sizes.^8^ ^,^ ^9^ While RVD by CTPA or echocardiography is an independent predictor of acute clinical deterioration,^10^ there have been inconsistent results regarding its relationship with 30-day mortality.^4^ ^,^ ^6^ ^,^ ^11^ ^–^ ^13^ Echocardiography studies have shown that as RVD severity increases, both risk of clinical deterioration and use of advanced interventions increase.^14^

We aimed to characterize the association of AI-derived CTPA cardiac measurements with in-hospital clinical deterioration (primary outcome) in a registry of patients with intermediate- to high-risk PE. The secondary objective was to compare retrospectively derived AI measurements in patients with or without use of advanced interventions (secondary outcome). For our exploratory objectives, we compared 1) radiologist vs AI-derived CTPA categorization of RV:LV and 2) AI vs echocardiography measurements. If, by retrospective study, we were to show that AI-derived CTPA measurements are strongly associated with acute clinical deterioration, then capturing immediately available CTPA cardiac measurements within clinical workflow could improve PE risk stratification.^15^

## METHODS

### Study Setting and Design

We conducted a retrospective analysis of data in our Clinical Outcomes Pulmonary Embolism Research Registry (COPERR). The COPERR is populated with adult patients identified as intermediate- or high-risk PE at presentation to any of eight Atrium Health emergency departments (ED) in North Carolina. We extracted data for registry patients who were treated between June 6, 2018–August 31, 2023. In November 2023, we requested a retrospective, remote AI analysis of CTPAs with confirmed index PE from this population of registry patients.

### Selection of Participants

Using the COPERR database, we identified adult patients (≥18 years) presenting to a participating ED who had 1) acute symptomatic PE as the primary ED diagnosis (by positive CTPA) and 2) intermediate- or high-risk PE classification. The PE risk was classified by emergency clinicians using European Society of Cardiology (ESC) guidelines^1^ and our PE response team’s (PERT) “Code PE” pathway (Supplemental Figure 1). The latter shows the structure, function, and logistics of PERT activation, triaging, multispecialty notification, and considerations for advanced PE interventions based on PE severity and bleeding risk. For the exploratory objective, we included above-mentioned patients with comprehensive transthoracic echocardiography (TTE) and RV-focused measurements completed within 24 hours of PE diagnosis.

We included patients with intermediate- or high-risk PE at ED presentation with CT images of 1-mm slice thickness available for AI analysis for the primary objective and with any AI analysis for the secondary objective. We excluded the following: patients with PE diagnosed only by high-probability ventilation/perfusion nuclear imaging; those whose point-of-care TTE findings were highly suspicious of PE but PE was not confirmed by CT; and those whose CTPA was not for index PE. We also excluded CTPAs that could not be analyzed by AI algorithm.

### Data Collection and Processing

Data entered in COPERR and available for analysis included demographics; clinical presentation features (including initial and worst vital signs within three hours of ED presentation); comorbidities; PE risk factors; criteria used for PE risk stratification; radiologist report of RV:LV; TTE measurements, dates, and times; PERT notification dates and times; laboratory measurements; PE-related outcomes and interventions; and adverse events.^14^ ^,^ ^16^ ^,^ ^17^ Trained data extractors retrieved information from the electronic health record and entered data in the registry.

During real-time clinical care of index PE hospitalization, RV:LV was measured by board-certified radiologists, and TTE was performed by certified cardiac sonographers from an echocardiography laboratory accredited by the Intersocietal Commission for the Accreditation of Echocardiography Laboratories. Given this was a retrospective study, the radiologists and sonographers were not aware of the study or its objectives. Radiologists measured RV:LV on the minor cardiac axis on CTPA. Measurements were at the widest points between the inner free wall of each ventricle to the inner wall of the ventricular septum. Radiologists used RV:LV cut-off of 1.0, with less than 1.0 considered negative for RV dilatation.

Sonographers used standard or RV-focused apical views to measure end-diastolic RV inner diameter at the base. The LV basal end-diastolic measurements were performed in the parasternal long axis view. Images were uploaded into a secure local server and portal system Merge Cardio (Merative LP, Ann Arbor, MI [formerly IBM Watson Health]). Board-certified cardiologists interpreted images and measurements and were blind to study and clinical outcomes. Only initial echocardiography measurements for index PE hospitalization were used in this study.

For each registry patient included in the study, we exported the fully anonymized digital imaging and communications in medicine (DICOM) file for each CTPA to share with the AI vendor for analysis. We transferred DICOM data from our study center to the server of an AI operating system (Aidoc, Tel Aviv, Israel) using encrypted secure file transfer protocol. Prior to transfer, all data were de-identified per the safe harbor de-identification protocol defined by the Health Insurance Portability and Accountability Act. The *de-identified accession number* was extracted from the DICOM header of shared studies. The study center used the key pair of *de-identified accessions* and *identified accessions* computed at the data anonymization step to re-identify data for the study.

The Aidoc PE algorithm is FDA cleared via the 510(k) premarket notification pathway required of all AI software medical devices. Aidoc’s use in detecting PE on CTPAs has been previously reported.^8^ ^,^ ^18^ The prototype of the PE detection algorithm was developed using input from anonymized, 1-mm series of CTPA reconstructions and based on a deep convolutional neural network comprising a Resnet architecture and trained and validated on over 25,000 CTPAs taken from many institutions. Aidoc algorithms had specific CTPA inclusion criteria, including slice thickness, kernel, and contrast phase to allow analysis. Aidoc has two software components: one for software analysis of CTPA DICOM files, and another for real-time analysis and reporting of interpretations to clinicians and radiologists. Only the first component was used in this study. The AI analyses of CTPAs and measurements were not performed during real-time clinical care.

Each CTPA was analyzed by two AI algorithms independently. For the first algorithm, if a PE was detected, AI determined whether the PE was a central clot or not. Central clot was defined by the following locations: pulmonary trunk; saddle (bifurcation of the main pulmonary artery trunk); right or left main pulmonary arteries or lobar pulmonary arteries. For the second algorithm, AI measured each RV and LV largest diameter (between inner walls) as a number and calculated the ratio of RV to LV. This was produced in a four-step process, including ventricular detection, ventricular segmentation, interventricular septum detection, and caliper positioning and measurements. The AI algorithm also identified patients with large central PEs. It is important to note a subsegmental PE did not provide a positive result. This was done to allow the AI-augmented clinical workflow to accurately identify acute PEs with RV dilatation as necessary conditions for intermediate- and high-risk PE classification.

The AI-based algorithm variables included the following categorical values: 1) Did the Aidoc algorithm analyze the data (yes or no); and 2) did the CTPA contain a PE (yes or no)? The AI-based continuous variables were RV basal diameter, LV basal diameter, and RV:LV. All data for AI-derived CTPA variables were matched to pertinent study IDs and uploaded into a standard electronic form within Research Electronic Data Capture (REDCap) tools at our institution.

### Outcomes

The primary outcome was PE-related clinical deterioration, defined as a composite of one or more of the following clinical deterioration events within days of index PE hospitalization: death; cardiac arrest; sustained hypotension treated with vasoactive medications; or rescue respiratory intervention (mechanical or positive pressure ventilation).^14^ The secondary outcome was use of advanced PE-specific interventions, including systemic thrombolysis, catheter-directed interventions, extracorporeal membrane oxygenation (ECMO), or surgical embolectomy.

### Statistical Analysis

Sample size was determined by the number of patients eligible for study analysis. To determine association with PE-related clinical deterioration (primary outcome), we used various statistical methods. We used bivariable analysis with the Student *t*-test or chi square to stratify by primary outcome groups. We conducted multivariable analyses for the primary outcome in two ways. First, we used least absolute shrinkage and selection operator (LASSO) regression to develop two models, one with AI assessment variables only and one with all independent variables. We reported missingness of each variable and used complete case analysis. We expressed strength of association as odds ratios with 95% confidence intervals (CI). Second, we used random forest (RF) to statistically infer the strength of the association of all independent variables in the dataset and identify the top 20 predictors of PE-related clinical deterioration (primary outcome) in a variable importance plot.

For each model’s prognostic performance on the primary outcome, we reported discrimination as area under the curve (AUC) and calibration as calibration plots with calibration statistics, including Brier, Brier scaled, intercept and slope. Performance for RF and LASSO logistic models was based on out-of-bag samples and 10-fold cross validation, respectively. Finally, to address the trade-off of false positives and false negatives, we used the Youden index to determine optimal RV:LV cut-offs and other AI-derived measurements for prognosis of clinical deterioration. For the selected optimal RV:LV and other AI cardiac measurements, we determined sensitivity, specificity, likelihood ratios, and AUC with 95% CI.

To determine association with the use of advanced interventions (secondary outcome), we used bivariable analysis with the Student *t*-test or chi square to stratify by secondary outcome groups. To measure reliability between AI-derived and radiologist CT classification of RV:LV ≥ 1.0 vs < 1.0, we used the Cohen kappa with its 95% CIs. We used suggested guidelines of Landis and Koch to describe the strength of agreement for the κ statistic: less than 0 = poor; 0 to 0.20 = slight; 0.021 to 0.40 = fair; 0.41 to 0.60 = moderate; 0.61 to 0.80 = substantial; and 0.81 to 1.00 = almost perfect.^19^

We reported mean and standard deviation time intervals in hours between PERT notification and TTE for the middle 95%. We used two methods to assess agreement between AI-derived CT cardiac and TTE measurements for RV, LV, and RV:LV. First, we used Pearson correlations with 95% CIs for continuous variables to test for magnitude and direction of linear relationships.^20^ Second, we used Bland-Altman plots to depict the relationship of difference and mean for each pair of CTPA and TTE measurements.

### Disclosures

Regarding the relationship with the company that developed and markets the AI-based PE algorithm used in this study, we declare that Aidoc had no role in the design of the study, the collection, analysis, and interpretation of data, or the preparation of the published manuscript. We further declare that we have not received and will not receive any compensation, direct or indirect, from Aidoc or any of its affiliates. We do not own stock in the company.

## RESULTS

### Study Flow

**Figure 1.**
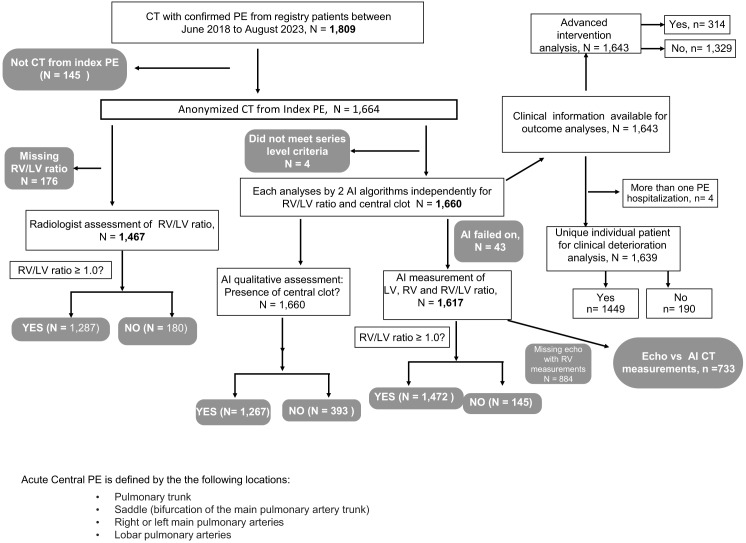
Study flow diagram*. *AI*, artifical intelligence; *PE*, pulmonary embolism; *CTPA*, computed tomography pulmonary angiography, *RV*, right ventricle; *LV*, left ventricle.

Figure 1 shows we screened 1,809 patients with CTPA-confirmed acute PE diagnosed in ED. Of these, 1,664 (92.0%) had CTPA associated with index PE diagnosis and anonymized DICOM files transferred for AI analysis. Radiologists provided categorical RV:LV classification for 1,467 of 1,664 (88.2%) CTPAs. The AI vendor analyzed 1,660 of the 1,664; four cases were excluded because of inadequate CTPA slice thickness for AI analysis. The AI assessment for central clot was successful in all (100%) CTPAs and 1,267 (76.3%) were found to have large central PE by the algorithm. The AI-derived cardiac measurements were obtained for 1,617/1,660 (97.4%). The AI failed to analyze 43 CTPAs because 1) they did not meet study inclusion criteria (i.e., slice thickness, kernel, contrast phase), or 2) the RV:LV algorithm was unable to detect appropriate landmarks to perform RV:LV analysis. Of 1,664 CTPAs, 733 (44.1%) had comprehensive TTE measurements during index PE hospitalization. Mean and SD for time interval between CTPA and TTE for the middle 95% was 13.6 (11.3) hours.

We were able to determine primary outcome responses for 1,639 unique patients **(** Table 1 **)** and secondary outcome for 1,643 unique patients. Of the 1,639, mean age was 63.0 ± 16 years, 805 (49.1%) were male, 997 (60.8%) were White, and 190 (11.6%) had one or more components of the primary outcome. Four patients had more than one ED visit for acute PE during the 2018–2023 study period. We reported PE-related clinical deterioration (primary outcome) for first visit only.

**Table 1.** Patient and imaging characteristics by pulmonary embolism-related clinical deterioration (primary outcome).

	Primary outcome (−) (n = 1,449)	Primary outcome (+) (n = 190)	Total N = 1,639	*P*-value
Age, years				
Mean (SD)	62.8 (16.0)	63.2 (15.4)	62 (15.9)	0.73
Race				
White	893 (61.6%)	104 (54.7%)	997 (60.8%)	0.41
Black	504 (34.8%)	81 (42.6%)	585 (35.7%)	
American Indian/Alaskan Native	12 (0.8%)	2 (1.1%)	14 (0.9%)	
Asian	5 (0.3%)	0 (0%)	5 (0.3%)	
Other	7 (0.5%)	0 (0%)	7 (0.4%)	
Pacific Islander/Native Hawaiian	1 (0.1%)	0 (0%)	1 (0.1%)	
Unknown	27 (1.9%)	3 (1.6%)	30 (1.8%)	
Sex				
Male	724 (50.0%)	81 (42.6%)	805 (49.1%)	0.06
Female	725 (50.0%)	109 (57.4%)	834 (50.9%)	
Ethnicity				
Non-Hispanic/Latino	1,347 (93.0%)	181 (95.3%)	1,528 (93.2%)	0.4
Hispanic/Latino	33 (2.3%)	3 (1.6%)	36 (2.2%)	
Unknown	69 (4.8%)	6 (3.2%)	75 (4.6%)	
Body surface area				
Mean (SD)	2.1 (0.328)	1.9 (0.338)	2 (0.331)	0.01
Intensive care unit admission				
No	759 (52.4%)	27 (14.2%)	786 (48.0%)	<0.001
Yes	686 (47.3%)	163 (85.8%)	849 (51.8%)	
Missing	4 (0.3%)	0 (0%)	4 (0.2%)	
Prior diagnosis of PE or DVT?				
No	1,118 (77.2%)	149 (78.4%)	1,267 (77.3%)	0.76
Yes	331 (22.8%)	41 (21.6%)	372 (22.7%)	
Family history of VTE?				
No	1,312 (90.5%)	180 (94.7%)	1,492 (91.0%)	0.07
Yes	128 (8.8%)	9 (4.7%)	137 (8.4%)	
Missing	9 (0.6%)	1 (0.5%)	10 (0.6%)	
Recent hospitalization (in 3 weeks)?				
No	1,260 (87.0%)	143 (75.3%)	1,403 (85.6%)	<0.001
Yes	187 (12.9%)	47 (24.7%)	234 (14.3%)	
Missing	2 (0.1%)	0 (0%)	2 (0.1%)	
Anticoagulation use?				
No	1,315 (90.8%)	170 (89.5%)	1,485 (90.6%)	0.60
Yes	131 (9.0%)	20 (10.5%)	151 (9.2%)	
Missing	3 (0.2%)	0 (0%)	3 (0.2%)	
Current or recent pregnancy (or miscarriage) within 6 weeks				
No	1,271 (87.7%)	173 (91.1%)	1,444 (88.1%)	0.96
Yes	12 (0.8%)	1 (0.5%)	13 (0.8%)	
Missing	166 (11.5%)	16 (8.4%)	182 (11.1%)	
Recent limb immobilization (current or within 3 weeks)				
No	1,381 (95.3%)	173 (91.1%)	1,554 (94.8%)	0.02
Yes	64 (4.4%)	16 (8.4%)	80 (4.9%)	
Missing	4 (0.3%)	1 (0.5%)	5 (0.3%)	
Recent trauma (in the prior 4–6 weeks)?				
No	1,417 (97.8%)	185 (97.4%)	1,602 (97.7%)	0.91
Yes	32 (2.2%)	5 (2.6%)	37 (2.3%)	
Surgery required (mechanical ventilation or epidural) within 6 weeks?				
No	1,323 (91.3%)	167 (87.9%)	1,490 (90.9%)	0.16
Yes	126 (8.7%)	23 (12.1%)	149 (9.1%)	
Clotting disorders (protein C, S, factor V deficiency)?				
No	1,394 (96.2%)	181 (95.3%)	1,575 (96.1%)	0.52
Yes	44 (3.0%)	8 (4.2%)	52 (3.2%)	
Missing	11 (0.8%)	1 (0.5%)	12 (0.7%)	
Hormone replacement therapy				
No	1,354 (93.4%)	179 (94.2%)	1,533 (93.5%)	0.80
Yes	95 (6.6%)	11 (5.8%)	106 (6.5%)	
Known pulmonary hypertension				
No	1,382 (95.4%)	179 (94.2%)	1,561 (95.2%)	0.59
Yes	67 (4.6%)	11 (5.8%)	78 (4.8%)	
Chronic pulmonary disease				
No	1,199 (82.7%)	147 (77.4%)	1,346 (82.1%)	0.08
Yes	250 (17.3%)	43 (22.6%)	293 (17.9%)	
Congestive heart failure				
No	1,333 (92.0%)	169 (88.9%)	1,502 (91.6%)	0.19
Yes	116 (8.0%)	21 (11.1%)	137 (8.4%)	
Total Charlson comorbidity index				
Mean (SD)	1.4 (2.16)	1.9 (2.38)	1 (2.20)	0.003
Median [min, max]	0 [0, 14.0]	1.0 [0, 9.00]	0 [0, 14.0]	
Lowest systolic BP (within 3 hours), mm Hg				
Mean (SD)	121 (21.8)	97.1 (27.0)	118 (23.7)	<0.001
Lowest O_2_ sat (within 3 hours), %				
Mean (SD)	93.1 (5.52)	85.5 (16.4)	92 (8.00)	<0.001
Missing	2 (0.1%)	1 (0.5%)	3 (0.2%)	
Highest HR (within 3 hours)				
Mean (SD)	106 (21.2)	120 (22.2)	108 (21.8)	<0.001
Median [min, max]	106 [11.0, 198]	121 [62.0, 178]	108 [11.0, 198]	
Highest RR (within 3 hours)				
Mean (SD)	24.4 (8.64)	31.3 (11.1)	25 (9.22)	<0.001
Median [min, max]	23.0 [14.0, 200]	30.0 [16.0, 103]	23 [14.0, 200]	
Missing	4 (0.3%)	2 (1.1%)	6 (0.4%)	
Shock index greater than 1.0?				
No	1,080 (74.5%)	69.0 (36.3%)	1,149 (70.1%)	0.01
Yes	179 (12.4%)	23.0 (12.1%)	202 (12.3%)	
Missing	190 (13.1%)	98 (51.6%)	288 (17.6%)	
Advanced/escalated PE intervention?				
No	1,233 (85.1%)	92 (48.4%)	1,325 (80.8%)	<0.001
Yes	216 (14.9%)	98 (51.6%)	314 (19.2%)	
Type of advanced intervention: systemic thrombolysis				
No	1,377 (95.0%)	127 (66.8%)	1,504 (91.8%)	<0.001
Yes	72 (5.0%)	63 (33.2%)	135 (8.2%)	
Catheter-directed intervention				
No	1,337 (92.3%)	173 (91.1%)	1,510 (92.1%)	0.65
Yes	112 (7.7%)	17 (8.9%)	129 (7.9%)	
Right ventricular assist device				
No	1,449 (100%)	190 (100%)	1,639 (100%)	NA
Yes	0 (0%)	0(0%)	0 (0%)	
ECMO				
No	1,449 (100%)	185 (97.4%)	1,634 (99.7%)	<0.001
Yes	0 (0%)	5 (2.6%)	5 (0.3%)	
Inferior vena cava filter used				
No	1,411 (97.4%)	177 (93.2%)	1,588 (96.9%)	0.17
Yes	34 (2.3%)	8.00 (4.2%)	42 (2.6%)	
Missing	4.0 (0.3%)	5.0 (2.6%)	9 (0.5%)	
Computed tomography assessment of CT by radiologists				
RV:LV < 1.0	157 (10.9%)	24.0 (12.6%)	181 (11.0%)	0.53
RV:LV ≥ 1.0	1,153 (79.8%)	147 (77.4%)	1,303 (79.5%)	
Missing	135 (9.3%)	19 (10%)	155 (9.5%)	
RV:LV (AI)				
RV:LV ≥ 1	1,115 (77.2%)	132 (69.5%)	1,250 (76.3%)	0.02
RV:LV < 1	330 (22.8%)	58 (30.5%)	389 (23.7%)	
RV basal width, by AI, cm				
Mean (SD)	5.2 (0.73)	5.3 (0.71)	5 (0.730)	0.02
Missing	20 (1.4%)	6 (3.2%)	26 (1.6%)	
LV basal width, by AI, cm				
Mean (SD)	3.8 (0.72)	3.6 (0.77)	3 (0.732)	<0.001
Missing	20 (1.4%)	6 (3.2%)	26 (1.6%)	
RV:LV ratio, by AI				
Mean (SD)	1.3 (0.324)	1.5 (0.39)	1 (0.336)	<0.001
Missing	20 (1.4%)	6 (3.2%)	26 (1.6%)	
Echocardiography RV basal width (ECHO)				
Mean (SD)	4.22 (0.811)	4.25 (0.814)	4 (0.812)	0.70
Missing	640 (44.2%)	110 (57.9%)	752 (45.9%)	
LV basal width (ECHO)				
Mean (SD)	4.1 (0.811)	3.9 (0.846)	4 (0.817)	0.004
Missing	153 (10.6%)	42 (22.1%)	196 (12.0%)	
RV:LV (ECHO)				
Mean (SD)	1.0 (0.272)	1.1 (0.332)	1 (0.278)	0.07
Missing	685 (47.3%)	116 (61.1%)	1 (0.278)	
RV:LV cut-off = 1.0 by cardiologist				
RV:LV ≥ 1.0	1,155 (79.7%)	147 (77.4%)	1,302 (79.4%)	0.72
RV:LV < 1.0	158 (10.9%)	24 (12.6%)	182 (11.1%)	
Missing	136 (9.4%)	19 (10%)	155 (9.5%)	
Abnormal troponin^*^	965 (66.6%)	163 (85.5%)	1,128 (68.8%)	<0.001
Initial troponin, ng/mL				
Mean (SD)	0.22 (1.45)	0.37 (0.92)	0.24 (1.4)	0.19
Missing	725 (50.0%)	98 (51.6%)	823 (50.2%)	
Initial high-sensitivity troponin, mean (SD), ng/mL	195 (606)	434 (1,420)	224 (756)	0.10
Missing	711 (49.1%)	88 (46.3%)	99 (48.17%)	

* We used troponin I or high-sensitivity troponin assays (Abbott, Abbott Park, IL) measured in ng/mL assay. Normal values for troponin I were less than 0.07 ng/mL. Normal values for high-sensitivity troponin were less than 12 for females and less than 20 for males. Abnormal troponin levels were higher than above-mentioned cut-offs.

*AI*, artificial intelligence algorithm; *CT*, computed tomography; *BP*, blood pressure; *DVT*, deep vein thrombosis; *ECHO*, echocardiography; *ECMO,* extracorporeal membrane oxygenation; *HR*, heart rate; *ng/mL*, nanograms per milliliter; *O_2_ sat*, oxygen saturation; *RR*, respiratory rate; *LV*, left ventricle; *RV*, right ventricle; *RV:LV*, right ventricle to left ventricle diameter ratio; *VTE*, venous thromboembolism.

### Patient Characteristics

There were no significant differences between those with or without clinical deterioration for age, gender, race, or ethnicity. There were significant differences for mean values of vital signs. Patients who had PE-related clinical deterioration (primary outcome) had lower systolic blood pressure and oxygen saturation readings and higher respiratory rate and heart rates than patients without clinical deterioration. There was significantly increased use of systemic thrombolysis, ECMO, and surgical embolectomy in the primary outcome group. However, there were no significant differences in use of catheter-directed interventions between outcome groups. For categorical cardiac CTPA assessments, Table 1 shows radiologists’ binary categorization of RVD using the RV:LV cut-off 1.0 was not significant between primary outcome groups. In contrast, AI-derived RV:LV binary categorization was significant. For mean AI-derived CTPA measurements, Table 1 shows significant differences in RV:LV, RV, and LV basal diameters between those with and without clinical deterioration.

For the 733 patients with TTE, TTE measurements were less than AI-derived CT cardiac measurements. Only LV basal diameter had significant differences between the primary outcome groups. Although mean RV basal diameter was above normal limits, the difference was not statistically significant for outcome-negative and outcome-positive groups.

### Primary Outcome

Multivariable analyses with unadjusted LASSO for PE-related clinical deterioration (primary outcome) showed the most significant independent AI-derived predictors were RV:LV (19.28 [3.0–109.4]) and central clot by AI (2.4 [1.6–3.6]). Both the adjusted LASSO and RF models vetted all candidate database variables. Both RF and adjusted LASSO prognostic models had excellent discrimination and calibration metrics for prognostic accuracy (Supplemental Figure 2): For discrimination, adjusted LASSO and RF had AUC of 0.88 (0.85, 0.90) and 0.87 (0.84, 0.89), respectively. Both models were well calibrated with Brier scores of 0.07. The RF model was slightly less calibrated than the LASSO model on other calibration metrics.

**Figure 2.**
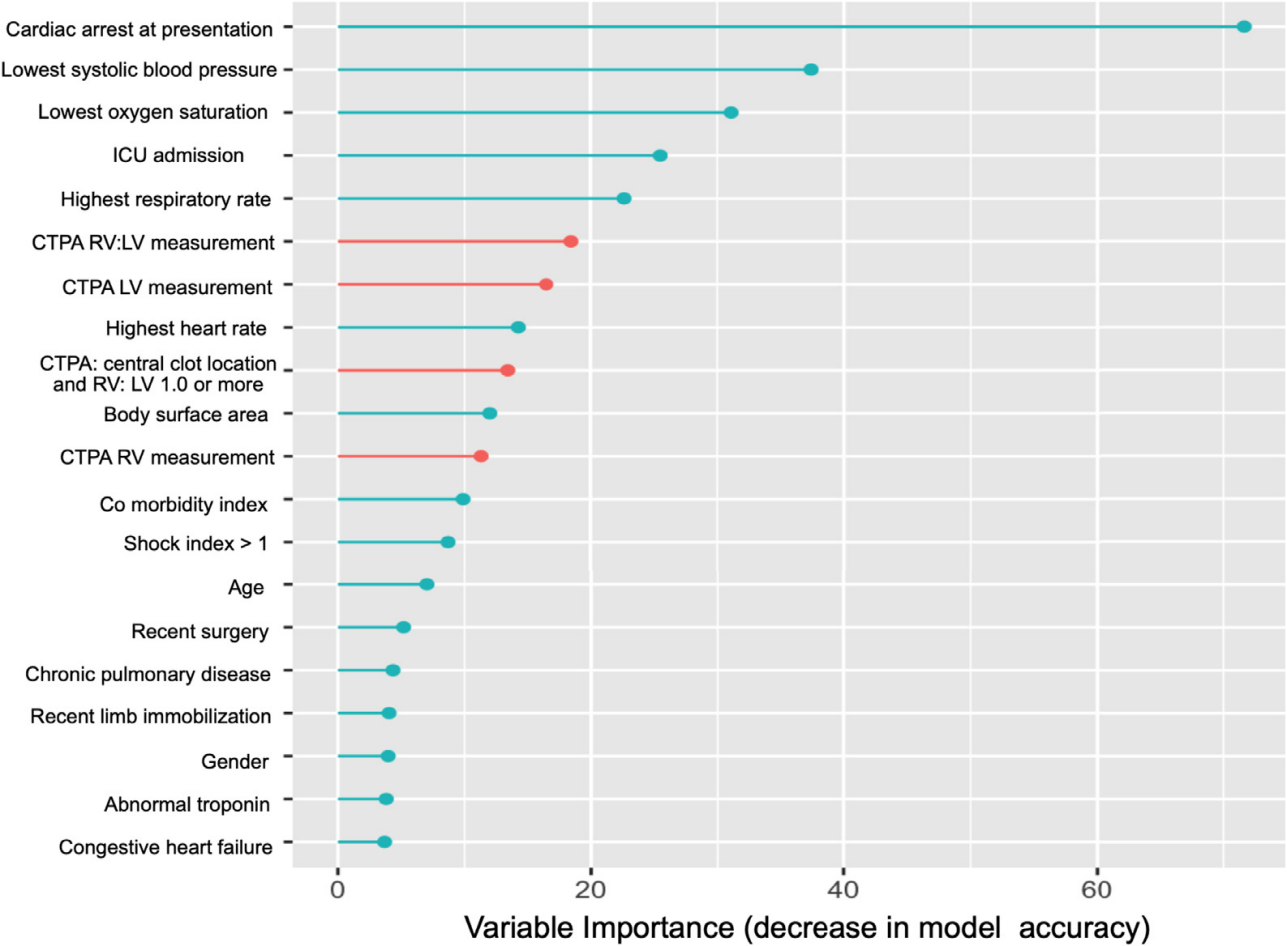
Random forest variable importance plot for predicting clinical deterioration. *CTPA*, computed tomography pulmonary angiography; *LV*, left ventricle; *RV*, right ventricle; *ICU*, intensive care unit.

Table 2 and Figure 2 show cardiac arrest at presentation was the top predictor of in-hospital clinical deterioration in both multivariable models (LASSO and RF). Admission to the intensive care unit, lowest systolic blood pressure, lowest oxygen saturation, and highest heart and respiratory rates were also top predictors in both models. The CTPA cardiac measurements were among the top 11 predictors selected by LASSO. Abnormal troponin was one of the top predictors by LASSO but had a lower influence on RF model accuracy than CTPA assessments. The CTPA cardiac measurements and findings of central clot location with RV:LV ≥ 1.0 were among the top 10 independent predictors of clinical deterioration in the RF model.

**Table 2.** LASSO^*^ regression models (unadjusted and adjusted) for pulmonary embolism-related clinical deterioration (primary outcome).

Unadjusted model with AI-derived CTPA assessments only			
	PE-related clinical deterioration (primary outcome)		
*Predictors*	*Odds ratios*	*Confidence interval*	*P-value*
RV:LV by AI	19.28	3.03–109.36	0.001
Central clot by AI	2.44	1.64–3.63	<0.001
RV diameter by AI	0.62	0.36–1.10	0.10
LV diameter by AI	1.46	0.68–2.93	0.31
Observations	1617		
R^2^ Tjur	0.046		
**Adjusted model with all variables considered**			
	**PE-related clinical deterioration (primary outcome)**		
***Predictors***	***Odds ratios***	***Confidence interval***	***P-value***
Initial cardiac arrest requiring CPR	97.6	29.14–462.2	<0.001
ICU admission	4.43	2.77–7.96	<0.001
Abnormal troponin	2.34	1.42–7.96	0.001
CTPA central clot location with RV:LV >1.0, determined by AI	2.08	1.30–3.31	0.002
Previous hospitalization within 3 weeks	1.71	1.05–2.73	0.03
Total Charlson comorbidity index	1.10	1.01–1.19	0.02
Highest respiratory rate within 3 hours of presentation	1.02	1.00–1.05	<0.001
Lowest systolic blood pressure within 3 hours of presentation	0.97	0.96–0.98	<0.001
Observations	1,617		
R^2^ Tjur	0.347		

* *LASSO*, least absolute shrinkage and selection operator; *PE*, pulmonary embolism; *AI*, artificial intelligence; *CTPA*, computed tomography pulmonary angiogram; *RV*, right ventricle; *LV*, left ventricle; *CPR*, cardiopulmonary resuscitation; *ICU*, intensive care unit.

Table 3 shows optimal cut-offs of AI-derived cardiac CTPA measurements with prediction metrics for PE-related clinical deterioration as RV:LV 1.54 (OR 2.5 [1.85–3.45] and AUC 0.6 [0.66, 0.70]). These cut-off values had high negative predictive values (NPV) but low positive predictive values (PPV).

**Table 3.** Optimal cut-offs of artificial intelligence-derived cardiac CTPA^*^ measurements with prediction metrics for pulmonary embolism-related clinical deterioration (primary outcome).

Variable	Cut-off	*P*-value	Sensitivity	Specificity	PPV	NPV	AUC	Odds ratio
RV:LV by AI	1.54	<0.001	0.52 (0.45, 0.59)	0.7 (0.67, 0.72)	0.18 (0.15, 0.22)	0.92 (0.9, 0.94)	0.63 (0.59, 0.68)	2.53 (1.85, 3.45)
RV diameter by AI, cm	5.30	0.03	0.57 (0.5, 0.64)	0.54 (0.51, 0.57)	0.14 (0.11, 0.16)	0.91 (0.89, 0.93)	0.56 (0.51, 0.6)	1.56 (1.15, 2.13)
LV diameter by AI, cm	4.02	<0.001	0.61 (0.54, 0.68)	0.62 (0.59, 0.64)	0.17 (0.14, 0.2)	0.93 (0.91, 0.94)	0.63 (0.59, 0.68)	2.58 (1.88, 3.54)

* *CTPA*, computed tomography pulmonary angiogram; *AI*, artificial intelligence; *cm*, centimeter; *PPV*, positive predictive value; *NPV*, negative predictive value; *AUC*, area under the curve; *RV*, right ventricle; *LV*, left ventricle.

### Secondary Outcome

Table 4 shows bivariable analysis of cardiac assessments stratified by use of advanced interventions (secondary outcome). Regardless of how cardiac measurements were derived, there were significant differences in cardiac measurements (whether continuous or categorical) between those with and without advanced interventions. For example, AI-derived CTPA RV:LV means with SDs were 1.62 (0.33) vs 1.35 (0.32) for those with and without advanced interventions (secondary outcome), respectively. With TTE, RV:LV means were 1.17 (0.29) vs 1.02 (0.27) for those with and without advanced interventions, respectively.

**Table 4.** Cardiac assessments grouped by use of advanced intervention (secondary outcome).

	No advanced intervention (n = 1,329)	Advanced intervention (n = 314)	Total N = 1,643	*P*-value
CT assessment by radiologist				
RV:LV ≥ 1.0	1,031 (77.6%)	274 (87.3%)	1,305 (79.4%)	<0.001
RV:LV < 1.0	165 (12.4%)	17 (5.4%)	182 (11.1%)	
Missing	133 (10%)	23 (7.3%)	156 (9.5%)	
CT assessments by AI				
RV:LV > 1 (AI)				
RV:LV > = 1	967 (72.8%)	286 (91.1%)	1,253 (76.3%)	<0.001
RV:LV < 1	362 (27.2%)	28 (8.9%)	390 (23.7%)	
RV basal width (AI)				
Mean (SD)	5.14 (0.729)	5.55 (0.633)	5.22 (0.729)	<0.001
Missing	19 (1.4%)	7 (2.2%)	26 (1.6%)	
LV basal width (AI)				
Mean (SD)	3.93 (0.732)	3.53 (0.642)	3.86 (0.73)	<0.001
Missing	19 (1.4%)	7 (2.2%)	26 (1.6%)	
RV:LV (AI)				
Mean (SD)	1.35 (0.316)	1.62 (0.332)	1.40 (0.34)	<0.001
Missing	19 (1.4%)	7 (2.2%)	26 (1.6%)	
ECHO assessments				
RV:LV > = 1	1,031 (77.6%)	274 (87.3%)	1,305 (79.4%)	<0.001
RV:LV < 1	165 (12.4%)	17 (5.4%)	182 (11.1%)	
Missing	133 (10.0%)	23 (7.3%)	156 (9.5%)	
Echocardiography				
LV diameter (AI)				
Mean (SD)	3.93 (0.73)	3.53 (0.64)	3.86 (0.73)	<0.001
Missing	19 (1.4%)	7 (2.2%)	26 (1.6%)	
RV basal width (ECHO)				
Mean (SD)	4.15 (0.778)	4.55 (0.895)	4.22 (0.81)	<0.001
Missing	587 (44.2%)	165 (52.5%)	752 (45.8%)	
LV basal width (ECHO)				
Mean (SD)	4.19 (0.842)	4.02 (0.676)	4.16 (0.82)	<0.001
Missing	149 (11.2%)	47 (15.0%)	196 (11.9%)	
RV:LV (ECHO)				
Mean (SD)	1.02 (0.269)	1.17 (0.293)	1.05 (0.28)	<0.001
Missing	627 (47.2%)	177 (56.4%)	804 (48.9%)	

*AI*, artificial intelligence algorithm; *CT*, computed tomography; *ECHO*, echocardiography; *LV*, left ventricle; *RV*, right ventricle; *RV:LV*, right ventricle to left ventricle diameter ratio.

### Exploratory Outcomes

There was agreement between AI and radiologists on RV:LV ≥ 1.0 for 1,224 cases and on RV:LV <1.0 for 67 cases (88% overall agreement [kappa 0.36, 95% CI 0.28–0.43], data not shown). The RV:LV means with SDs were 1.48 (0.31) and 0.86 (0.11), respectively. There was disagreement for 178 (12.1%) cases. RV:LV means were 1.23 (0.23) and 0.92 (0.05) when AI reported abnormal RV:LV vs RV:LV < 1.0, respectively. For comparison of AI-derived CTPA with TTE measurements, Pearson correlation coefficients for RV, LV, and RV:LV were 0.47 (0.42, 0.52), 0.58 (0.53, 0.62), and 0.50 (0.45, 0.55), respectively. All kappas were interpreted as moderate agreement per Landis and Koch guidelines. Supplemental Figure 3 shows strong negative bias with lower TTE measurements than CTPA measurements at presentation.

## DISCUSSION

We found AI-derived RV:LV measurements on CTPA were significantly greater in PE patients experiencing clinical deterioration or receiving advanced intervention than those without these outcomes. There was significantly increased use of systemic thrombolysis, ECMO, and surgical embolectomy in the primary outcome group. In our models, which had strong discrimination and calibration, AI-derived RV:LV measurements were independent predictors of clinical deterioration, along with abnormal vital signs and cardiac arrest at presentation in one or both multivariable models. The optimal RV:LV cut-off of 1.5 had an odds ratio of 2.5 and AUC of 0.6 for PE-related clinical deterioration (primary outcome). The AI-derived RV:LV measurements performed better as predictors of primary and secondary outcomes than radiologists’ or AI-derived categorizations using RV:LV cut-off of 1.0.

Other reports have focused on outcomes similar to ours. Beigel et al. performed a study evaluating 179 intermediate-risk PE patients for predictors of short-term death and advanced interventions.^21^ Twenty-six patients required advanced intervention, which was significantly associated with echocardiographic evidence of severe RVD (42% vs 19%, *P* < 0.01) or higher RV:LV measurement on CTPA (1.9 ± 0.6 vs 1.46 ± 0.5, *P* < 0.001). The RV dilatation on TTE was an independent predictor for advanced interventions. This information further corroborates the importance of measurements to risk stratify PE patients. Unlike TTE measurements, cardiac CTPA measurements are immediately available at the time of PE diagnosis for risk stratification.^22^

Other studies that assessed how CTPA cardiac measurements are associated with clinical outcomes had mixed results. A retrospective study by Foley et al. involving 101 patients with CT-proven PEs of any severity at a single center showed strong agreement (intraclass correlation 0.83, [0.77–0.88]) between radiologists’ and AI-derived CTPA measurements for RV:LV.^15^ In this study, RV:LV ranged from 0.67–2.43, with 65% being ≥ 1.0. The optimal RV:LV cut-off for 30-day mortality was 1.18. The use of AI analysis in our study led to a change in risk stratification in 45% of patients. However, in a large prospective study of 1,950 CT-confirmed PEs by Beenen et al., RV:LV measurements by radiologists were not significantly different between those with and without short-term mortality.^23^ Similar to the Foley et al. study, we found an elevated RV:LV had a strong association with in-hospital clinical deterioration in our intermediate- and high-risk PE cohort. Our optimal RV:LV cut-off of 1.5 was higher than theirs.

A previous report showed fair agreement (kappa 0.4) for categorical assessments of RV dysfunction between CTPA and TTE.^22^ Our study found moderate agreement of RV:LV measurements by CTPA and TTE. We believe our findings underscore the importance of using immediately available CTPA measurements of RVD for risk stratification and prognosis. However, at many institutions, RV measurements are not routinely performed or interpreted on CTPA. One study in a large regional healthcare system with 21 sites showed only 18.3% of 1,571 positive CTPA interpretation reports included RV measurements.^24^

The use of AI to detect PE and analyze CTPA cardiac measurements at time of PE presentation may improve risk stratification for PERTs and provide quality assurance to enhance radiologists’ workflow. The diagnostic accuracy of AI should include a low number of false positives to minimize notification fatigue and potential for medication mismanagement. In a retrospective multicenter study, Cheik et al. evaluated diagnostic performances of the Aidoc PE algorithm on CTPAs and compared them with those of radiologists to determine impact of AI PE detection.^18^ Of 1,202 patients included, the AI algorithm detected 219 suspicious PEs, of which 176 were true PEs, including 19 true PEs missed by radiologists. The highest sensitivity and NPVs were obtained with AI, while the highest specificity and PPV were found with radiologists. Our retrospective study focused on less subtle PE diagnoses; the AI analysis was specifically created to focus on non-segmental PE, and AI agreed that PE findings were present in all CTPAs. Artificial intelligence further analyzed ventricle measurements on CTPA and determined central vs non-central filling defects.

Although our comparison of CTPA RV:LV categorization by AI vs radiologists had 88% agreement, the kappa 0.34 is interpreted as fair agreement. Agreement was more likely when RV:LV was well above or well below the 1.0 cut-off; the two sources were more likely to disagree when RV:LV was closer to 1.0. It is unknown whether AI-derived CTPA measurements might “correct” radiologist assessments in real time for those close to the 1.0 cut-off or whether such a “correction” would have clinical significance on patient care and outcomes. Even with an optimal RV:LV cut-off of 1.5, we note the low PPV for PE-related clinical deterioration. So, an RV:LV cut-off of 1.5 is not sufficient to be the sole determinant of decision-making about disposition or advanced interventions. Similar to another report, our study showed a combination of CTPA parameters (central clot location and RV:LV) had stronger associations with clinical deterioration than RV:LV alone (categorical or continuous).^22^

Incorporation of CTPA cardiac measurements in PE risk stratification may impact local/regional clinical practice or guidelines. Next steps may include prospective studies that include CTPA measurements as predictors of clinical outcomes and PERT risk stratification, and pragmatic comparisons of AI-assisted workflow vs traditional workflow in which CTPA cardiac measurements, clinical management metrics, and patient-centered outcomes are assessed.

## LIMITATIONS

Our study had several limitations. First, we conducted a retrospective, remote AI analysis of CTPA with confirmed intermediate- and high-risk PE. We did not study real-time AI analyses on recently completed CTPAs. Our study design and inclusion criteria, therefore, do not lend to any interpretation about diagnostic accuracy of the AI platform on CT of patients with lower acuity PE or without PE. We cannot report on false positive or false negative interpretations, potential impact on PERT notifications or clinical management, or compare to previous reports of AI’s diagnostic accuracy for PE. Theoretically, we have shown AI-derived measurements were better predictors of acute clinical deterioration than categorical radiologist assessment of RV:LV cut-off of 1.0. However, to show the impact of AI on patient care by clinicians, there would need to be pragmatic, randomized controlled trials comparing usual care vs AI-assisted clinical care. Prospective studies would enable reporting timeliness of AI analysis of CT and its effect on radiologist workload, physician notification of positive and significant findings, and impact of measurements on risk assignment, resource utilization, advanced interventions, and clinical deterioration.

Other limitations are specific to the exploratory objectives. Our study did not verify whether agreements between radiologist and AI for RV:LV ≥ 1.0 were correct; both interpretations could be incorrect. Study design could be improved by including a comparator, such as a reference standard (e.g., cardiac magnetic resonance imaging), use of an independent, blinded radiologist for separate measurements or to serve as an adjudicator, or earlier contemporaneous TTE measurements. For the second exploratory objective, we did not determine presence or absence of interventions in the interval between CT and TTE. The TTE and CTPA were performed at different times and often more than 12 hours apart. Therefore, the differences between these measured variables may be due to worsening or improving cardiac burden during the time intervals. Not all patients in the cohort had TTE. High missingness of TTE measurements was a limitation in comparison of them with the AI-derived CTPA measurements. The differences observed in these mean measurements may be due to different imaging modality or time interval between studies. The subgroup that had TTE likely represented those with higher acuity at presentation.

## CONCLUSION

Right ventricle:left ventricle measurements of 1.5 or more on the initial CT pulmonary angiogram had strong associations with in-hospital clinical deterioration and advanced interventions in a large database of intermediate- and high-risk patients with pulmonary embolism. This study points to the potential of capitalizing on immediately available CTPA RV:LV measurements for gauging PE severity and for risk stratification.

## Supplementary Information






