# Supplementary material for: Cardiac Computed Tomography Measurements in Pulmonary Embolism Associated with Clinical Deterioration
Source: West J Emerg Med. 2025 Jan 15;26(2):219–32. doi: 10.5811/westjem.20763 (PMC11931709; doi:10.5811/westjem.20763)
Supplement: Supplementary file 1 [file wjem-26-219-s001.pdf]

Atrium Health

Code Pulmonary Embolism Pathway

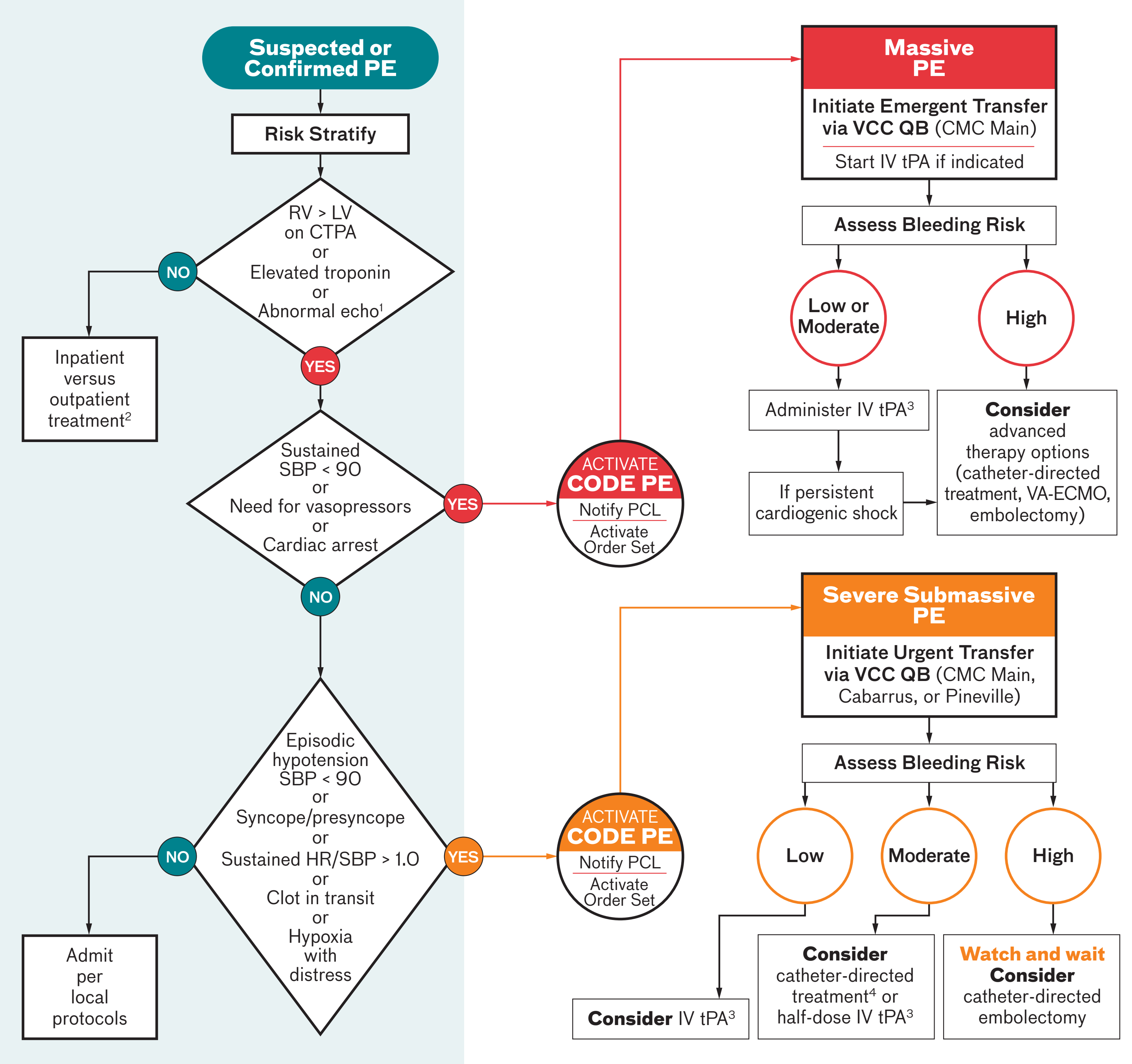

High Bleeding Risk:

- Gastrointestinal bleeding within previous 30 days
- Life threatening hemorrhage in any of the following sites at the time of diagnosis: intraperitoneal, retroperitoneal, pulmonary, uterine, bladder, or nose
- Head trauma causing loss of consciousness within previous 7 days
- Any history of hemorrhagic stroke
- Ischemic stroke within the past year
- History of intraocular hemorrhage
- Known or suspected intracranial metastasis
- Known inherited or acquired bleeding disorder, e.g., hemophilia, platelet count < 50,000/uL, or liver failure with prothrombin time abnormal (INR > 1.7)
- Surgery that required opening of the chest cavity, peritoneum, skull or spinal canal within the previous 14 days
- Pregnancy
- Large pericardial effusion

Moderate Bleeding Risk:

- Age > 65
- Dementia
- Surgery > 14 days but < 60 days
- Any prior stroke or symptoms suggesting transient ischemic attack in the past 30 days
- Any prior gastrointestinal bleeding
- Current use of thienopyridine (clopidogrel, prasugrel, ticagrelor), direct thrombin inhibitors (dabigatran) or factor Xa inhibitors (rivaroxaban, apixaban)
- INR > 1.7 from warfarin use
- Any metastatic cancer, recent tongue bite, recent fracture, recent fall with head strike
- History of hematuria or frequent nosebleeds
- Severe uncontrolled hypertension on initial presentation (SBP > 185 mmHg or DBP > 110 mmHg)

Abbreviations:

PE: pulmonary embolism, **PCL:** physician connection line, **RV:** right ventricle, **LV:** left ventricle, **CTPA:** computed tomography pulmonary angiogram, **SBP:** systolic blood pressure, **HR:** heart rate, **VCC QB:** virtual critical care quarterback, **tPA:** tissue plasminogen activator

Annotations:

1. **Abnormal echo findings include:** dilated and/or hypokinetic RV, septal flattening or bowing
2. Refer to Outpatient Management of VTE diagnosed in the Emergency Department protocol
3. If immediate IV thrombolysis or catheter-directed treatment anticipated, prefer UFH administration**
4. Includes both cather-directed thrombolysis and embolectomy

**Stop heparin drip during administration of tPA. Recheck PTT or anti-Xa immediately after tPA infusion is complete. As long as PTT < 111 seconds or anti-Xa < 0.7, restart heparin drip WITHOUT bolus at same rate as before tPA was started.
