# Supplementary figures and images for: Cardiac Computed Tomography Measurements in Pulmonary Embolism Associated with Clinical Deterioration
Source: West J Emerg Med. 2025 Jan 15;26(2):219–32. doi: 10.5811/westjem.20763 (PMC11931709; doi:10.5811/westjem.20763)

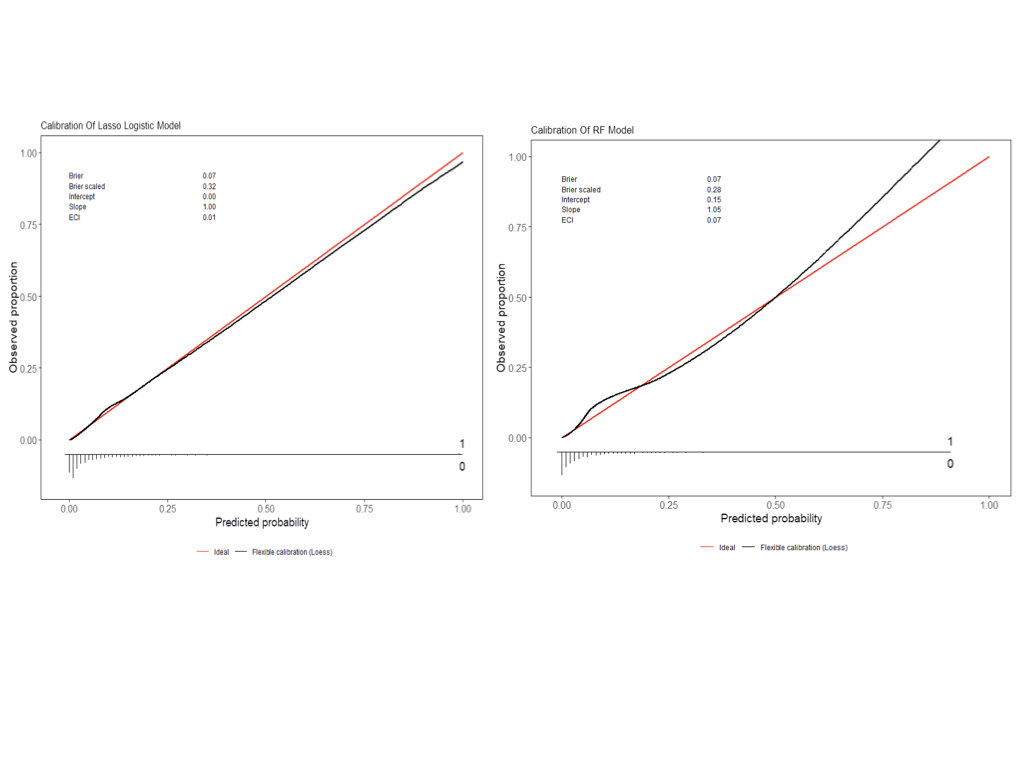

Supplement: Supplementary file 2 [file wjem-26-219-s002.png]

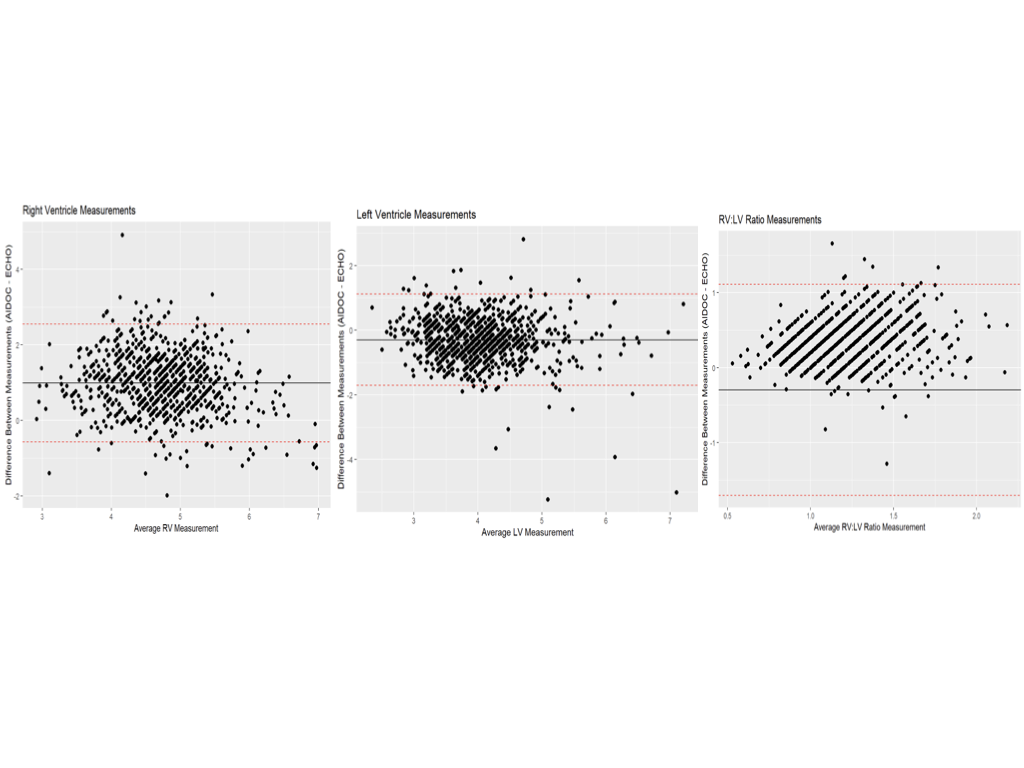

Supplement: Supplementary file 3 [file wjem-26-219-s003.png]
